# Kinesiophobie bei Schulterbeschwerden: Validierung der deutschen Version der Tampa Scale for Kinesiophobia (TSK-GV)

**DOI:** 10.1007/s00482-022-00678-2

**Published:** 2022-12-02

**Authors:** Larissa Pagels, Kerstin Lüdtke, Axel Schäfer

**Affiliations:** 1 Institut für Gesundheitswissenschaften, Universität zu Lübeck, Ratzeburger Allee 160, 23562 Lübeck, Deutschland; 2 Hochschule für angewandte Wissenschaften und Kunst (HAWK) Hildesheim, Goschentor 1, 31134 Hildesheim, Deutschland

**Keywords:** Tampa Scale, Schulter, TSK, Schulterschmerzen, Validität, Tampa scale, Shoulder, TSK, Shoulder pain, Validity

## Abstract

**Hintergrund:**

Schulterbeschwerden bilden mit einer Prävalenz von 7 bis 30 % die drittgrößte muskuloskeletale Beschwerdegruppe. Ihre Entstehung und Entwicklung wird u. a. durch psychische Faktoren beeinflusst. Die Tampa Scale for Kinesiophobia (TSK) ist international das gängigste Messinstrument zur Erhebung bewegungsbezogener Angst.

**Ziel der Arbeit:**

Untersuchung der Reliabilität und Validität der TSK-GV in einer Population mit Schulterbeschwerden.

**Material und Methoden:**

Im Rahmen einer multizentrischen Querschnittsstudie wurden Proband*innen mit Schulterschmerzen eingeschlossen. Es wurden neben der Kinesiophobie die Schmerzintensität, die subjektive Beeinträchtigung im täglichen Leben sowie die Angst-Vermeidungs-Überzeugungen erfasst.

**Ergebnisse:**

Insgesamt konnten 49 Proband*innen (24 Frauen und 25 Männer) mit einem mittleren Alter von 41,8 Jahren (SD = 12,8) eingeschlossen werden. Die deskriptive Auswertung auf Itemebene zeigte eine gute interne Konsistenz (Cronbachs *α* = 0,81). Die Homogenität der Skala ist schwach (Loevingers *H* = 0,35). Die Korrelationsberechnungen ergaben keine deutliche Konvergenz der TSK-GV mit dem FABQ (*r* = 0,3501; *p* = 0,0137). Die divergente Validität konnte sowohl zur NRS (*r* = 0,1216; *p* = 0,4052) als auch zum SPADI (*r* = 0,2571; *p* = 0,0745) bestätigt werden. Die Hypothesentestung ergab 28,57 % angenommener Hypothesen. Es zeigte sich ein signifikanter Einfluss der TSK-GV und des FABQ auf die Beschwerdedauer (*R*^*2*^ = 0,3652; *p* ≤ 0,0001) sowie eine erklärte Varianz der Beschwerdedauer auf die TSK-GV von *R*^*2*^ = 0,1834 (*p* = 0,0021). Die Subgruppenanalyse zeigte einen signifikant höheren Grad der Kinesiophobie bei den männlichen Probanden (*t* = 3,8084/*df* = 47; *p* = 0,0002).

**Diskussion:**

Die TSK-GV ist ein reliables Messinstrument. Die Konstruktvalidität sollte in zukünftigen Studien weiter untersucht werden. Die Ergebnisse dieser Studie zeigen vergleichbare Werte zu vorangegangenen Studien in anderen Populationen. Die TSK-GV ist das bislang einzige validierte deutschsprachige Messinstrument zur Erhebung der bewegungsbezogenen Angst bei Schulterbeschwerden und zeigt akzeptable Werte für diese Population.

**Zusatzmaterial online:**

Die Online-Version dieses Beitrags (10.1007/s00482-022-00678-2) enthält den Fragebogen „Tampa Scale for Kinesiophobia (TSK-GV)“.

## Abstract (en)

**Background:**

With a prevalence of 7–30%, shoulder disorders form the third largest group of musculoskeletal complaints. Their formation and development is influenced by, e.g. psychological factors. The Tampa scale for kinesiophobia (TSK) is the most common measure for quantifying fear of movement.

**Objectives:**

To investigate the reliability and validity of the German version of the TSK (TSK-GV) in a shoulder pain population.

**Materials and methods:**

Subjects with shoulder pain were included in a multicenter cross-sectional study. In addition to kinesiophobia, pain intensity, subjective impairment in daily life and fear-avoidance beliefs were recorded.

**Results:**

A total of 49 subjects (24 women and 25 men) with a mean age of 41.8 (SD = 12.8) were included. The descriptive evaluation at item level showed good internal consistency (Cronbach’s *α* = 0.81). The homogeneity of the scale is poor (Loevinger’s *H* = 0.35). The correlations did not show any clear convergence of the TSK-GV with the fear-avoidance beliefs questionnaire (FABQ) (*r* = 0.3501; *p* = 0.0137). The divergent validity was confirmed for both the numerical rating scale (NRS) (*r* = 0.1216; *p* = 0.4052) and the shoulder pain and disability index (SPADI) (*r* = 0.2571; *p* = 0.0745). The hypothesis testing resulted in 28.57% accepted hypotheses. There was a significant influence of the TSK-GV and the FABQ on the duration of complaints (*R*^*2*^ = 0.3652; *p* ≤ 0.0001), as well as an explained variance of the duration of complaints on the TSK-GV of R^2^ = 0.1834 (*p* = 0.0021). The subgroup analysis showed a significantly higher degree of kinesiophobia in male subjects (*t* = 3.8084/*df* = 47; *p* = 0.0002).

**Conclusion:**

The TSK-GV is a reliable measurement tool. The construct validity should be further investigated in future studies. This study shows comparable values to previous studies in other populations. The TSK-GV is to date the only validated German language measure for recording fear of movement in shoulder disorders and shows an acceptable fit for this population.

## Hintergrund

Schulterbeschwerden bilden nach Rückenschmerzen und Nackenschmerzen die drittgrößte Gruppe der muskuloskeletalen Beschwerden [7]. Die globale Prävalenz variiert von 7 bis 30 % [3]. Schulterbeschwerden stellen damit sowohl in Deutschland als auch in vergleichbaren Ländern weltweit ein erhebliches Gesundheitsproblem dar, welches nicht nur individuelles Leid für die Betroffenen, sondern auch wirtschaftliche und gesellschaftliche Folgen nach sich zieht [1]. 2019 waren Patient*innen mit Schulterläsionen in Deutschland für 18,69 Tage pro 100 Versicherungsjahre arbeitsunfähig [23]. Allein diese Diagnose machte 4,5 % des Leistungsvolumens von krankengymnastischen Verordnungen der AOK aus und stand damit an Platz zwei der häufigsten Diagnosen mit Verordnungen von Krankengymnastik [24]. Schulterbeschwerden haben häufig einen langwierigen Verlauf, wobei nur 60 % der Betroffenen innerhalb von einem Jahr vollständig genesen [21]. Gerade chronische Schulterschmerzen können selten durch eine Gewebeverletzung vollständig erklärt werden [10], vielmehr sollten psychologische Einflussfaktoren berücksichtigt werden [14]. Bewegungsbezogene Angst stellt dabei einen der häufigsten Gründe für Funktionseinschränkungen dar [4]. Die bewegungsbezogene Angst beinhaltet die Dimensionen Angst-Vermeidungs-Überzeugung, Angst vor Bewegung und Kinesiophobie [5]. Letztere wird als übermäßige, irrationale und einschränkende Angst vor körperlicher Bewegung und Aktivität beschrieben und resultiert aus einem Gefühl der Gefahr für schmerzhafte (erneute) Verletzungen. Ergebnisse einer Studie zeigen, dass über 50 % der Patient*innen, welche sich in einer physiotherapeutischen Praxis vorstellten, eine signifikante Kinesiophobie aufwiesen [6]. Da Kinesiophobie einen negativen Einfluss auf die Rehabilitationsdauer und das Therapieergebnis nach einer Verletzung hat, ist es wichtig, Kinesiophobie standardisiert messen zu können und die Ergebnisse in die Behandlungsplanung einzubeziehen. Es ist anzunehmen, dass Betroffene mit Kinesiophobie von einem rein biomedizinischen Behandlungsansatz nur eingeschränkt profitieren. Ein frühzeitiges Erkennen spielt daher für den Behandlungserfolg eine wichtige Rolle [6]. Die Tampa Scale for Kinesiophobia (TSK) ist international das gängigste Messinstrument zur Erhebung von bewegungsbezogener Angst [11]. Sie wurde 2014 von Rusu et al. in die deutsche Sprache übersetzt und für Patient*innen mit Kreuzschmerzen validiert [16]. Um die TSK-GV auch bei Patient*innen mit Schulterbeschwerden einsetzen zu könne, müssen die Gütekriterien in dieser Population untersucht werden.

## Studiendesign und Untersuchungsmethoden

Die TSK-GV ist ein „patient-reported outcome measure“ (PROM) mit insgesamt elf Items und zwei Subskalen: somatischer Fokus und Aktivitätsvermeidung. Sie wird anhand einer 4‑Punkt-Likert-Skala beantwortet und es kann eine maximale Punktzahl von 44 erreicht werden, was der größtmöglichen Kinesiophobie entspricht. Den Fragebogen finden Sie im Online-Zusatzmaterial.

Die Studie ist eine multizentrische Querschnittsstudie. Die Proband*innenrekrutierung fand von Januar bis Juni 2021 in acht physiotherapeutischen Praxen in Schleswig-Holstein, Hamburg und Niedersachsen statt.

Hierfür wurden die folgenden Inklusionskriterien formuliert: Alter > 65 Jahre, Shoulder-Pain-and-Disability-Index(SPADI)-Score von mind. 20 % und ausreichende Deutschkenntnisse (Verständnis der Fragen). Proband*innen wurden exkludiert, wenn sie ein frisches Trauma (< 5 Tage) im Bereich der Schulter erlitten hatten oder neurologische Erkrankungen als Ursache für die Schulterbeschwerden infrage kamen.

Erhebungsinstrumente waren ein Kurzfragebogen mit soziodemografischen Fragen sowie eine numerische Rating-Skala (NRS; 11 Punkte) zur Einschätzung der aktuellen Schmerzintensität. Die funktionellen Beeinträchtigungen im Alltag wurden mit dem SPADI [1] erhoben. Der Fear-Avoidance Beliefs Questionnaire (FABQ; [13]) und die TSK-GV [16] wurden erhoben, um bewegungsbezogene Angst in den Dimensionen Angst-Vermeidungs-Überzeugungen und Kinesiophobie zu erheben.

Es wurde eine deskriptive Auswertung der soziodemografischen Daten und der TSK-GV auf Itemebene durchgeführt. Hierbei wurde auch die interne Konsistenz ermittelt sowie die konvergente und divergente Validität auf Itemebene analysiert. Die Güte der Konstruktvalidität wurde neben der Strukturvalidität auch durch eine Hypothesentestung bestimmt. Die Hypothesen wurden bezüglich der Korrelationen der TSK-GV zu den weiteren erhobenen Messinstrumenten sowie zu Unterschieden in einer Subgruppenanalyse aufgestellt. Des Weiteren wurden mittels multipler linearer Regressionsmodelle Zusammenhänge der TSK-GV mit weiteren Variablen analysiert.

## Fallzahl

Zur Analyse der konvergenten und divergenten Validität der TSK-GV wurde eine Fallzahl von *n* = 47 angestrebt mit der Erwartung einer Korrelation von r < 0,4 (Power 0,5) für die divergente Validität und r > 0,75 (Power 0,7) für die konvergente Validität. Eine adäquate Fallzahl für die linearen Regressionsmodelle wurde für R^2^ = 0,2 mit drei Kovariablen mit *n* = 48 berechnet.

## Ethik

Die Studie wurde von der Kommission für Forschungsethik der Hochschule für angewandte Wissenschaften und Kunst (HAWK) Hildesheim am 21.12.2020 zugelassen. Die Proband*innen wurden schriftlich über die Ziele, Inhalte und Risiken der Studie aufgeklärt und konnten jederzeit ohne Angabe von Gründen abbrechen. Alle Proband*innen haben sich schriftlich zur Teilnahme bereiterklärt.

## Ergebnisse

### Beschreibung der Stichprobe und Messergebnisse

Es wurden entsprechend den Einschlusskriterien 49 Proband*innen rekrutiert. Zum Messzeitpunkt betrug die Schmerzintensität im Mittel 3,88 (SD 2,02). Die Beeinträchtigungen im Alltag wurden mit einem SPADI-Score von 41,32 % (SD 15,57 %) bewertet. Der FABQ wurde im Mittel mit *M* = 26,43 (*SD* = 18,21) und die TSK-GV mit *M* = 23 (*SD* = 6,37) bewertet (Tab. 1). Höhere Werte in den Messinstrumenten bedeuten jeweils eine größere Ausprägung des abgebildeten Konstrukts [1, 13, 16].

**Table 1:** 

Stichprobe	*n* = 49
Alter, *M (SD)*	41,8 (12,8)
Geschlecht weiblich, *n (%)*	24 (48,98)
Operiert, *n (%)*	13 (26,53)
Zeit seit Op. in Wochen, *M (SD)*	9,23 (5,6)
Beschwerdedauer in Monaten, *M (SD)*	32,55 (83,26)
Chronisch, *n (%)*	31 (63,27)
Zuvor PT, *n (%)*	16 (32,65)
Sport, *n (%)*	42 (85,71)
Zeit für Freizeitsport in h/Woche, *M (SD)*	4,86 (3,09)
Leistungssportler*innen, *n (%)*	8 (16,33)
Zeit für Wettkampfsport in h/Woche, *M (SD)*	7,88 (5,49)
NRS, *M (SD)*	3,88 (2,02)
SPADI, *M (SD)*	41,32 (15,57)
FABQ, *M (SD)*	26,43 (18,21)
TSK-GV, *M (SD)*	23 (6,37)

*n* Anzahl, *%* Prozentzahl, *M* Mittelwerte, *SD* Standardabweichung

### Deskriptive Auswertung der TSK-GV auf Itemebene

Die Verteilung der Antworten auf die einzelnen Antwortkategorien sowie die Ergebnisse zur internen Konsistenz und Homogenität der TSK-GV zeigt Tab. 2. Das Cronbachs *α* für die TSK-GV beträgt *α* = 0,81, für die Subskala TSK-SF *α* = 0,71 und für die TSK-AA *α* = 0,72. Ein Ausschluss einzelner Items kann das Cronbachs *α* der Skala im Bereich von 0,61 bis 0,75 verändern. Im Allgemeinen wird ein Cronbachs *α* von > 0,8 für die interne Konsistenz eines PROM als gut und von > 0,7 als adäquat angesehen [20]. Die interne Konsistenz der TSK-SF und der TSK-AA ist somit als adäquat zu bewerten und die Item-Skala-Konsistenz als schwach bis adäquat. Der Loevinger-*H*-Koeffizient beschreibt die Homogenität der Skala und liegt für die TSK-GV bei *H* = 0,35 und für die Subskala TSK-SF bei *H* = 0,34 und bei der TSK-AA bei *H* = 0,41. Der Loevinger-*H*_*j*_-Skalierbarkeitskoeffizient zeigt die Homogenität der Items an und zeigt an, dass ein Weglassen des Items 6 „*Schmerz bedeutet immer, dass ich mich verletzt habe*“ (*H*_*j*_ = 0,18) das Cronbachs *α* der TSK-AA steigern würde. Die Items der TSK-GV haben, mit Ausnahme des Items 6, eine schwache (*H*_*j*_ ≤ 0,4) bis moderate (*H*_*j*_ ≤ 0,5) Skalierbarkeit [18]. Item 5 hat den höchsten Loevinger-Skalierbarkeitskoeffizienten mit *H*_*j*_ = 0,56, welcher mit *H*_*j*_ > 0,5 als hoch bewertet wird ([18]; Tab. 2).

**Table 2:** 

Item	Antwortkategorie in %				Alpha-Item	Loev. *H*_*j*_-Koeff.	*n* nicht signifikanter *H*_*j*_*k-*Koeff.
	Überhaupt nicht einverstanden (1)	Mehr oder weniger nicht einverstanden (2)	Mehr oder weniger einverstanden (3)	Völlig einverstanden (4)			
*1*	38,78	22,45	22,45	16,33	0,68	0,37	1
*2*	12,24	28,57	30,61	28,57	0,70	0,38	1
*3*	55,10	24,49	14,29	6,12	0,61	0,46	0
*4*	71,43	16,33	10,20	2,04	0,70	0,30	2
*5*	81,63	10,20	6,12	2,04	0,61	0,56	0
*6*	40,82	30,61	18,37	10,20	0,75	0,18	4
*7*	34,69	14,29	26,53	24,49	0,66	0,40	1
*8*	53,06	22,45	22,45	2,04	0,65	0,43	1
*9*	6,12	12,24	51,02	30,61	0,69	0,37	1
*10*	51,02	10,20	26,53	12,24	0,66	0,40	0
*11*	24,49	32,65	14,29	28,57	0,68	0,34	1

*TSK-GV* Cronbachs *α* = 0,81, *H* = 0,34, *TSK-SF* Cronbachs *α* = 0,71, *H* = 0,41, *TSK-AA* Cronbachs *α* = 0,72, *H* = 0,35

### Konstruktvalidität

#### Strukturvalidität

Für die konvergente Itemvalidität konnte gezeigt werden, dass 8/11 Items (72,7 %) einen Korrelationskoeffizienten von *r* > 0,4 mit der Subskala, der sie zugeordnet werden, haben. Zudem weisen 9/11 Items (81,8 %) einen größeren Korrelationskoeffizienten mit der eigenen Subskala auf als mit der anderen, womit die divergente Validität auf Itemebene beschrieben wird. Damit wird die Bedingung der divergenten Validität, aber nicht die der konvergenten Validität auf Itemebene erfüllt ([12]; Tab. 3).

**Table 3:** 

Item	1	2	3	4	5	6	7	8	9	10	11
TSK-SF	*0,434*	***0,391***	*0,574*	***0,337***	*0,588*	0,143	0,416	0,564	0,418	0,453	0,340
TSK-AA	0,493	0,396	0,474	0,258	0,333	***0,227***	*0,539*	*0,576*	*0,425*	*0,535*	*0,456*

*TSK-SF* Tampa Scale for Kinesiophobia Somatic Focus, *TSK-AA* Tampa Scale for Kinesiophobia Activity Avoidance

#### Hypothesentestung

Die TSK-GV wurde zur Berechnung der konvergenten Validität mit dem FABQ und seinen drei Subskalen einzeln korreliert. Den höchsten Korrelationskoeffizienten zeigte die Korrelation von TSK-GV und FABQ‑3 mit *r* = 0,479 (*p* *≤* 0,001), was einem moderaten Zusammenhang entspricht [17].

Zur Ermittlung der divergenten Validität wurden Korrelationen der TSK-GV mit der NRS sowie mit dem SPADI berechnet. Hierbei ergab sich ein nicht signifikanter schwacher Zusammenhang zwischen der TSK-GV und der NRS (*r* = 0,122; *p* = 0,405; [17]). Die TSK-GV korreliert mit *r* = 0,257 (*p* = 0,075) mit dem SPADI-Gesamtwert, was als moderater Zusammenhang beschrieben werden kann ([17]; Tab. 4).

**Table 4:** 

	TSK	TSK-SF	TSK-AA	FABQ	FABQ‑1	FABQ‑2	FABQ‑3	NRS	SPADI	SPADI-Schmerz
*TSK*	**1**	**–**	**–**	**–**	**–**	**–**	**–**	**–**	**–**	**–**
*TSK-SF*	**0,86** ≤ 0,0001	**1**	**–**	**–**	**–**	**–**	**–**	**–**	**–**	**–**
*TSK-AA*	**0,92** ≤ 0,0001	**0,59** ≤ 0,0001	**1**	**–**	**–**	**–**	**–**	**–**	**–**	**–**
*FABQ*	**0,35** 0,01	**0,4** ≤ 0,01	0,25 0,08	**1**	**–**	**–**	**–**	**–**	**–**	**–**
*FABQ‑1*	0,22 0,13	0,21 0,14	0,18 0,21	**0,84** ≤ 0,0001	**1**	**–**	**–**	**–**	**–**	**–**
*FABQ‑2*	0,15 0,29	0,26 0,07	0,04 0,77	**0,88** ≤ 0,0001	**0,72** ≤ 0,0001	**1**	**–**	**–**	**–**	**–**
*FABQ‑3*	**0,48** ≤ 0,001	**0,49** ≤ 0,001	**0,38** ≤ 0,01	**0,67** ≤ 0,0001	0,27 0,06	**0,37** ≤ 0,01	**1**	**–**	**–**	**–**
*NRS*	0,12 0,41	0,05 0,71	0,15 0,30	0,18 0,21	0,04 0,79	0,03 0,83	**0,38** ≤ 0,01	**1**	**–**	**–**
*SPADI*	0,26 0,07	0,20 0,17	0,25 0,08	**0,44** ≤ 0,01	0,18 0,22	**0,43** ≤ 0,01	**0,46** ≤ 0,001	**0,45** ≤ 0,01	**1**	**–**
*SPADI-Schmerz*	**0,32** 0,03	**0,32** 0,03	0,26 0,07	**0,28** 0,05	0,06 0,7	0,21 0,14	**0,43** ≤ 0,01	**0,56** ≤ 0,0001	**0,77** ≤ 0,0001	**1**
*SPADI-Behinderung*	0,13 0,37	0,07 0,65	0,16 0,29	**0,44** ≤ 0,01	0,21 0,16	**0,47** ≤ 0,001	**0,38** ≤ 0,01	0,27 0,06	**0,88** ≤ 0,0001	**0,4** ≤ 0,01

*TSK* Tampa Scale for Kinesiophobia, *TSK-SF* TSK-Somatic Focus, *TSK-AA* TSK Activity Avoidance, *FABQ* Fear Avoidance Beliefs Questionnaire, *FABQ‑1* work pain beliefs, *FABQ‑2* work, *FABQ‑3* physical acitivity, *NRS* numerische Ratingskala, *SPADI* Shoulder and Pain Disability Index

In der Subgruppenanalyse konnte allein beim Vergleich der männlichen und weiblichen Teilnehmenden ein signifikanter Unterschied festgestellt werden. Die männlichen Probanden zeigten einen höheren TSK-GV-Wert (*t* = 3,808/*df* = 47, *p* *≤* 0,001).

Die Konstruktvalidität kann mit 28,57 % angenommener Hypothesen nicht als gut bewertet werden ([15]; Tab. 5).

**Table 5:** 

Korrelationen der TSK-GV zu Vergleichsfragebögen	Erwartetes Ergebnis	Ergebnis	Konnte die Hypothese angenommen werden?
TSK-GV vs. FABQ	Sehr starke Korrelation (r > 0,75)	Moderate Korrelation (*r* = 0,3501; *p* = 0,0137)	Nein
TSK-GV vs. SPADI	Schwache bis moderate Korrelation (*r* ≤ 0,4)	Moderate Korrelation (*r* = 0,2571; *p* = 0,0745)	Ja
TSK-GV vs. NRS	Schwache bis moderate Korrelation (*r* ≤ 0,4)	Schwache Korrelation (*r* = 0,1216; *p* = 0,4052)	Ja
*Subgruppenanalyse*			
Geschlecht	TSK-GV-Werte der Frauen sind signifikant (*p* ≤ 0,05) höher	Werte der Männer sind signifikant höher	Nein
Alter	TSK-GV-Werte der 40- bis 65-Jährigen sind signifikant (*p* ≤ 0,05) höher	Kein signifikanter Unterschied	Nein
Beschwerdedauer	Signifikanter Unterschied (*p* ≤ 0,05) der TSK-GV-Werte zwischen chronischen und akuten Proband*innen	Kein signifikanter Unterschied	Nein
Leistungssportler*innen	TSK-GV-Werte der Leistungssportler*innen sind signifikant (*p* ≤ 0,05) höher	Kein signifikanter Unterschied	Nein
*Wie viele Hypothesen konnten angenommen werden?*	–	*–*	*2/7 (28,57* *%)*

*TSK-GV* Tampa Scale for Kinesiophobia German Version, *FABQ* Fear Avoidacne Beliefs Questionnaire, *SPADI* Shoulder and Pain Disability Index, *NRS* numerische Ratingskala

### Regression

Für die Berechnung der Einflussfaktoren auf die TSK-GV (Primäranalyse) wurden die Prädiktoren Beschwerdedauer, Alter, SPADI und FABQ eingeschlossen, da dieses Modell im Zuge der Vorwärtsselektion die höchste erklärte Varianz mit *adj. R*^*2*^ = 0,20 aufwies, wobei lediglich die Variable Beschwerdedauer mit *t* = 2,73 und *p* = 0,009 einen statistisch signifikanten Einfluss auf die TSK-GV zeigt.

Im Sinne einer Sekundäranalyse wurde ermittelt, wie verschiedene Prädiktoren die Dauer der Schulterbeschwerden beeinflussen, indem verschiedene Regressionsmodelle aufgestellt wurden. Mit einem *adj. R*^*2*^ von 0,22 zeigte das Modell, welches die Variablen TSK-GV, FABQ, SPADI und NRS einschloss, den besten Wert. Das Regressionsmodell zeigt eine erklärte Varianz von *R*^*2*^ = 0,285 bei einer Signifikanz von *p* = 0,005. Hier zeigten die Prädiktoren TSK-GV (*t* = 2,5; *p* = 0,016) und FABQ (*t* = 2,13; *p* = 0,038) einen statistisch signifikanten Einfluss auf die Beschwerdedauer. Die Analyse der prognostischen Faktoren zeigte eine moderate Korrelation zwischen der TSK-GV und dem FABQ auf. Daraufhin wurde ein Regressionsmodell aufgestellt, welches die Interaktion der TSK-GV mit dem FABQ berücksichtigt. Dieses Regressionsmodell weist eine erklärte Varianz von *R*^*2*^ = 0,365 bei einer Signifikanz von *p* ≤ 0,0001 auf. Der Prädiktor TSK-GV + FABQ ist mit *t* = 5,2 (*p* ≤ 0,0001) ein eindeutig signifikanter Einflussfaktor für die Dauer der Beschwerden der Proband*innen (Tab. 6).

**Table 6:** 

Abhängige Variable	Prädiktoren	*R*^*2*^	*p*
*TSK-GV*	Beschwerdedauer, Alter, SPADI, FABQ	0,2649	0,0078
	Beschwerdedauer	0,1834	0,0021
*Beschwerdedauer*	TSK-GV, FABQ, SPADI, NRS	0,2854	0,0045
	TSK, FABQ	0,3652	0,0000

*TSK-GV* Tampa Scale for Kinesiophobia German Version

## Diskussion

Die Reliabilität der TSK-GV kann in Bezug auf die interne Konsistenz als gut bewertet werden. Aus der deskriptiven Auswertung der Skala lässt sich ableiten, dass über den Verbleib des Items 6 in der Skala sowie über eine Umstrukturierung der Subskalen nachgedacht werden sollte, um die interne Konsistenz der Skala zu erhöhen. Im Bereich der Konstruktvalidität entsprachen die Ergebnisse nicht den a priori formulierten Hypothesen, dies lässt auf eine geringe Konstruktvalidität schließen. Die Ergebnisse der Berechnungen zur Strukturvalidität werten diese allerdings auf. Interessant ist der deutliche Zusammenhang zwischen der Beschwerdedauer und der Kinesiophobie, wobei eine längere Beschwerdedauer einer höheren Kinesiophobie entspricht (s. Tab. 6). Diese Erkenntnis konnte in vorangegangenen Studien mit Patient*innen mit Schulterbeschwerden bislang nicht gewonnen werden.

Die Ergebnisse der Studie zeigen auf, dass die TSK-GV insgesamt ein passendes Messinstrument zur Erhebung der Kinesiophobie bei Patient*innen mit Schulterbeschwerden darstellt. Die Ergebnisse sind insgesamt vergleichbar mit denen vorangegangener Studien, bei welchen die Gütekriterien der TSK-GV sowie die der englischsprachigen TSK-11 untersucht wurden [8, 16, 19]. Im Vergleich zur Studie von Rusu et al. (2014 [16]), welche die TSK-GV bei einer Population mit lumbalen Kreuzschmerzen (engl. „low back pain“ [LBP]; *n* = 191) untersuchten, war die Stichprobe dieser Studie deutlich kleiner. Die Teilnehmenden dieser Studie waren im Schnitt 8,3 Jahre jünger und wiesen eine im Mittel um 3,49 Jahre kürzere Beschwerdedauer auf. Die Schmerzintensität, gemessen mit der NRS, war bei beiden Studien vergleichbar (LBP: *M* = 3,2; Schulter: *M* = 3,88). Ebenso die Höhe der Kinesiophobie (LBP: *M* = 21,34; Schulter:* M* = 23). Rusu et al. errechneten ein Cronbachs *α* von 0,73 für die Gesamtskala (TSK-SF: 0,64; TSK-AA: 0,63), was unter den in dieser Studie erlangten Werten liegt und aussagt, dass die TSK-GV die Kinesiophobie bei Patient*innen mit Schulterbeschwerden reliabler misst als bei Patient*innen mit LBP. Die Korrelationen der TSK-GV mit dem FABQ zeigten, übereinstimmend mit den Ergebnissen dieser Studie, auch bei Rusu et al. nur moderate Zusammenhänge mit beiden Skalen. Es ist allerdings fraglich, ob der Zusammenhang von Kinesiophobie mit Schulterbeschwerden vergleichbar mit dem Zusammenhang bei Rückenschmerzen ist. Diese Annahme wäre grundlegend dafür, die zuvor beschriebenen Studienergebnisse vergleichen zu können [22].

Zukünftig wäre eine weitere Untersuchung der Gütekriterien der TSK-GV insbesondere bei weiteren Populationen, z. B. Nackenschmerzen, sinnvoll. Untersuchungen zur Test-Retest-Reliabilität, zur Kriteriumsvalidität und zur Änderungssensitivität der TSK-GV wären mögliche nächste Schritte. Die vorliegenden Ergebnisse zeigen, dass die TSK-GV ein adäquates Messinstrument zur Erhebung der Kinesiophobie bei Patient*innen mit Schulterbeschwerden sein könnte.

Der Einfluss psychischer Faktoren wird in der Praxis stärker berücksichtigt, daher sollten diese im Praxisalltag erhoben werden und sich die Forschung zunehmend in diese Richtung entwickeln [6, 14]. Die Ergebnisse dieser Studie zeigen, dass die Beschwerdedauer signifikant mit dem Grad der Kinesiophobie zusammenhängt. Ein gezieltes Adressieren von Kinesiophobie, bspw. durch Edukation oder „behaviour change techniques“ (BCT), könnte sich positiv auf das Therapieergebnis der Betroffenen auswirken. Hierbei könnte auch ein interdisziplinärerer Ansatz (z. B. Physiotherapie und Psychotherapie) berücksichtigt werden, um die Betroffenen bestmöglich betreuen zu können, wie es bereits in der Schmerztherapie vermehrt gemacht wird [2].

## Limitationen

Limitierend ist zu sagen, dass die Stichprobe dieser Studie mit *n* = 49 zwar die a priori berechnete Fallzahl abdeckte, von der Consensus-based-Standards-for-the-Selection-of-Health-Status-Measurement-Instruments(COSMIN)-Checkliste aber bei allen durchgeführten Berechnungen als „doubtful“ beschrieben wird, eine Stichprobengröße von *n* ≥ 50 wird als adäquat bezeichnet [9]. Dieser Wert wird daher knapp verfehlt. Eine größere Stichprobe würde die Aussagekraft der Ergebnisse dieser Studie stärken. Des Weiteren ist der Einsatz des FABQ als Referenzscore zu diskutieren, da dieser bislang nicht für die Population mit Schulterbeschwerden validiert wurde und dies als Voraussetzung für die Berechnung der konvergenten Validität gilt [20]. Bislang liegt allerdings keine deutsche Version eines Messinstruments vor, welches ein mit der TSK-GV vergleichbares Konstrukt misst [11].

## Schlussfolgerung

Die TSK-GV ist aktuell das einzige Messinstrument zur Erhebung der bewegungsbezogenen Angst bei Patient*innen mit Schulterbeschwerden im deutschsprachigen Raum. Die TSK-GV ist ein ökonomisches Messinstrument mit akzeptablen Gütekriterien und eignet sich somit für den Einsatz in Therapie und Forschung. Das Erheben der Kinesiophobie sollte in der klinischen Entscheidungsfindung der Behandelnden berücksichtigt werden, um spezifische Maßnahmen einzuleiten [7]. Um die TSK-GV in anderen Populationen einsetzen zu können, bedarf es weiterer Forschung. Zukünftig wird zudem eine Ermittlung der Gütekriterien des FABQ durchgeführt.

## Fazit für die Praxis

- Der Einsatz der TSK-GV ist als Assessment im Rahmen der Therapie geeignet.
- Bei Vorliegen einer Kinesiophobie sollten weitere Disziplinen eingebunden werden.
- Die TSK-GV kann zu einer zielgerichteten patient*innenzentrierten Behandlung beitragen.
- Die TSK-GV hat das Potenzial, die Prognose von langwierigen Schulterbeschwerden zu schärfen.

### Supplementary Information





## Abkürzungen

COSMIN: Consensus-based Standards for the Selection of Health Measurement Instruments
FABQ‑1: FABQ-Subskala „work pain beliefs“
FABQ‑2: FABQ-Subskala „work“
FABQ‑3: FABQ-Subskala „physical activity“
LBP: „Low back pain“ (dt.: Kreuzschmerz)
MZP: Messzeitpunkt
PROM: „Patient-reported outcome measurements“
TSK-AA: TSK-Subskala „activity avoidance“
TSK-SF: TSK-Subskala „somatic focus“
