# Supplementary material for: Kinesiophobia in shoulder disorders: Validation of the German version of the Tampa scale for kinesiophobia (TSK-GV)
Source: Schmerz. 2022 Dec 2;38(3):198–204. [Article in German] doi: 10.1007/s00482-022-00678-2 (PMC11116245; doi:10.1007/s00482-022-00678-2)
Supplement: Supplementary file 1 [file 482_2022_678_MOESM1_ESM.pdf]

**Tampa Scale for Kinesiophobia (TSK-GV)**

Mit den nachfolgenden Fragen möchten wir untersuchen, wie Sie selbst zu Ihren Schmerzen stehen. Bitte geben Sie an, in welchem Maße Sie mit den vorgegebenen Aussagen einverstanden sind.

A = überhaupt nicht einverstanden
B = mehr oder weniger nicht einverstanden
C = mehr oder weniger einverstanden
D = völlig einverstanden

| Aussage | A | B | C | D |
|---|---|---|---|---|
| 1. Ich habe Angst davor, dass ich mich möglicherweise verletze, wenn ich Sport treibe. | | | | |
| 2. Wenn ich versuchen würde, mich über die Schmerzen hinweg zu setzen, würde sie noch schlimmer. | | | | |
| 3. Mein Körper sagt mir, dass ich etwas sehr Schlimmes habe. | | | | |
| 4. Mein Gesundheitszustand wird von anderen nicht ernst genug genommen. | | | | |
| 5. Wegen des Schmerzproblems ist mein Körper für den Rest meines Lebens gefährdet. | | | | |
| 6. Schmerz bedeutet immer, dass ich mich verletzt habe. | | | | |
| 7. Die sicherste Art, zu verhindern, dass meine Schmerzen schlimmer werden, ist einfach darauf zu achten, dass ich keine unnötigen Bewegungen mache. | | | | |
| 8. Ich hätte nicht so viele Schmerzen, wenn nicht etwas Bedenkliches in meinem Körper vor sich hinge. | | | | |
| 9. Meine Schmerzen sagen mir, wann ich mit dem Training aufhören muss, um mich nicht zu verletzen. | | | | |
| 10. Ich kann nicht all die Dinge tun, die gesunde Menschen machen, da ich mich zu leicht verletzen könnte. | | | | |
| 11. Niemand sollte Sport treiben müssen, wenn er/sie Schmerzen hat. | | | | |
